# Role of the Sympathetic Nervous System and Its Modulation in Renal Hypertension

**DOI:** 10.3389/fmed.2018.00082

**Published:** 2018-03-29

**Authors:** Yusuke Sata, Geoffrey A. Head, Kate Denton, Clive N. May, Markus P. Schlaich

**Affiliations:** ^1^Neurovascular Hypertension and Kidney Disease Laboratory, Baker Heart and Diabetes Institute, Melbourne, VIC, Australia; ^2^Faculty of Medicine, Nursing and Health Sciences, Central Clinical School, Monash University, Melbourne, VIC, Australia; ^3^Neuropharmacology Laboratory, Baker Heart and Diabetes Institute, Melbourne, VIC, Australia; ^4^Cardiovascular Program, Department of Physiology, Monash Biomedicine Discovery Institute, Monash University, Melbourne, VIC, Australia; ^5^Preclinical Critical Care Unit, The Florey Institute of Neuroscience and Mental Health, The University of Melbourne, Parkville, VIC, Australia; ^6^Dobney Hypertension Centre, School of Medicine – Royal Perth Hospital Unit, University of Western Australia, Perth, WA, Australia

**Keywords:** sympathetic nervous system, renal nerve activity, hypertension, renal, central nervous system, afferent

## Abstract

The kidneys are densely innervated with renal efferent and afferent nerves to communicate with the central nervous system. Innervation of major structural components of the kidneys, such as blood vessels, tubules, the pelvis, and glomeruli, forms a bidirectional neural network to relay sensory and sympathetic signals to and from the brain. Renal efferent nerves regulate renal blood flow, glomerular filtration rate, tubular reabsorption of sodium and water, as well as release of renin and prostaglandins, all of which contribute to cardiovascular and renal regulation. Renal afferent nerves complete the feedback loop *via* central autonomic nuclei where the signals are integrated and modulate central sympathetic outflow; thus both types of nerves form integral parts of the self-regulated renorenal reflex loop. Renal sympathetic nerve activity (RSNA) is commonly increased in pathophysiological conditions such as hypertension and chronic- and end-stage renal disease. Increased RSNA raises blood pressure and can contribute to the deterioration of renal function. Attempts have been made to eliminate or interfere with this important link between the brain and the kidneys as a neuromodulatory treatment for these conditions. Catheter-based renal sympathetic denervation has been successfully applied in patients with resistant hypertension and was associated with significant falls in blood pressure and renal protection in most studies performed. The focus of this review is the neural contribution to the control of renal and cardiovascular hemodynamics and renal function in the setting of hypertension and chronic kidney disease, as well as the specific roles of renal efferent and afferent nerves in this scenario and their utility as a therapeutic target.

## Introduction

The sympathetic innervation of the kidneys has drawn increasing scientific and clinical interest over the last decade, particularly, after the introduction of catheter-based renal sympathetic denervation into clinical medicine demonstrated marked reductions in blood pressure in patients with resistant hypertension (1, 2). Renal sympathetic nerves play a key role in blood pressure regulation and play a crucial role in the pathogenesis of hypertension, a condition commonly characterized by substantially elevated renal sympathetic nerve activity (RSNA) (3).

Increased RSNA has been demonstrated to contribute to the rise in blood pressure through three major mechanisms which include: (1) an increase in tubular reabsorption of urinary sodium and water, (2) a reduction of renal blood flow and glomerular filtration rate (GFR), and (3) release of renin from the juxtaglomerular apparatus, thereby activating the renin–angiotensin–aldosterone cascade (4). Sustained sympathetic overactivity has been associated with the development of end organ damage such as cardiac hypertrophy, deterioration of kidney function, and others. It is, therefore, not surprising that efforts aimed at exploiting the therapeutic potential of neuromodulation have been widely investigated, culminating in the clinical application of renal denervation, which has recently been proven to be effective in lowering blood pressure in a sham-controlled study in drug-naive hypertensive patients.

The aim of this article is to summarize the evidence of from recent scientific reports to assess the utility and future potential of therapeutically targeting the neural control of kidney in cardiovascular and renal disease.

## Sympathetic Innervation of the Kidney

### Efferent Renal Nerves—Anatomy

The initial evidence for the presence of functional renal sympathetic innervation was based on the observation of a change in urine volume following denervation and stimulation of renal nerves in anesthetized animals (5). Since surgical transplantation of the kidney was described by Carrel and Guthrie (6), extensive animal studies revealed the key roles of renal nerves on modulation of renal vascular tone and excretory function (7).

While inter-species and inter-animal variations exist, major neural structures that supply nerve fibers to the kidneys include the celiac plexus, lumbar splanchnic nerves, and intermesenteric plexus. The celiac plexus consists of the aorticorenal ganglion, celiac ganglion, and major splanchnic nerves (8). Since hypertension induced by renal artery constriction or irradiation of the kidneys is not affected by excision of extrinsic renal nerves, these nerves are not considered responsible in the pathogenesis of renal hypertension (9).

On the other hand, efferent intrinsic innervation of the kidney is distributed along the renal artery and vein, which subsequently branch around the arterial vascular segments in the renal cortex and outer band of medulla (10). Although the renal efferent nerves densely innervate the cortex and outer medulla, innervation is also evident around the vascular smooth muscle cells in the afferent and efferent arterioles and along the inner medulla. Therefore, stimulation of renal nerves primarily reduces blood flow in the outer cortex, with high levels of stimulation also reducing flow in the medulla.

Sympathetic innervation of the renal vasculature is adrenergic and most pronounced around terminal varicosities and the juxtamedullary region of the inner cortex, as neuroeffector junctions (11). The release of norepinephrine in the kidney has been demonstrated by an arteriovenous gradient and increased norepinephrine concentration in urine, whereas the decrease in renal norepinephrine release induced by renal nerve denervation is supported by the observation of a dramatic reduction of norepinephrine content in renal tissue of up to 95% (12).

In all adrenergic sympathetic postganglionic nerve terminals, dopamine, a precursor of norepinephrine is also present. In response to renal sympathetic nerve stimulation, both norepinephrine and dopamine are released from adrenergic sympathetic postganglionic nerve terminals (4). Given that renal function in response to renal nerve stimulation is not altered by administration of dopamine antagonists, dopamine is considered to play only a marginal role as a functional neurotransmitter in the kidney. Recently, gene expression studies of dopamine receptors revealed the distribution and functional mechanisms of dopamine in the kidneys (13, 14).

### Efferent Renal Nerves—Function

**Figure 1:**
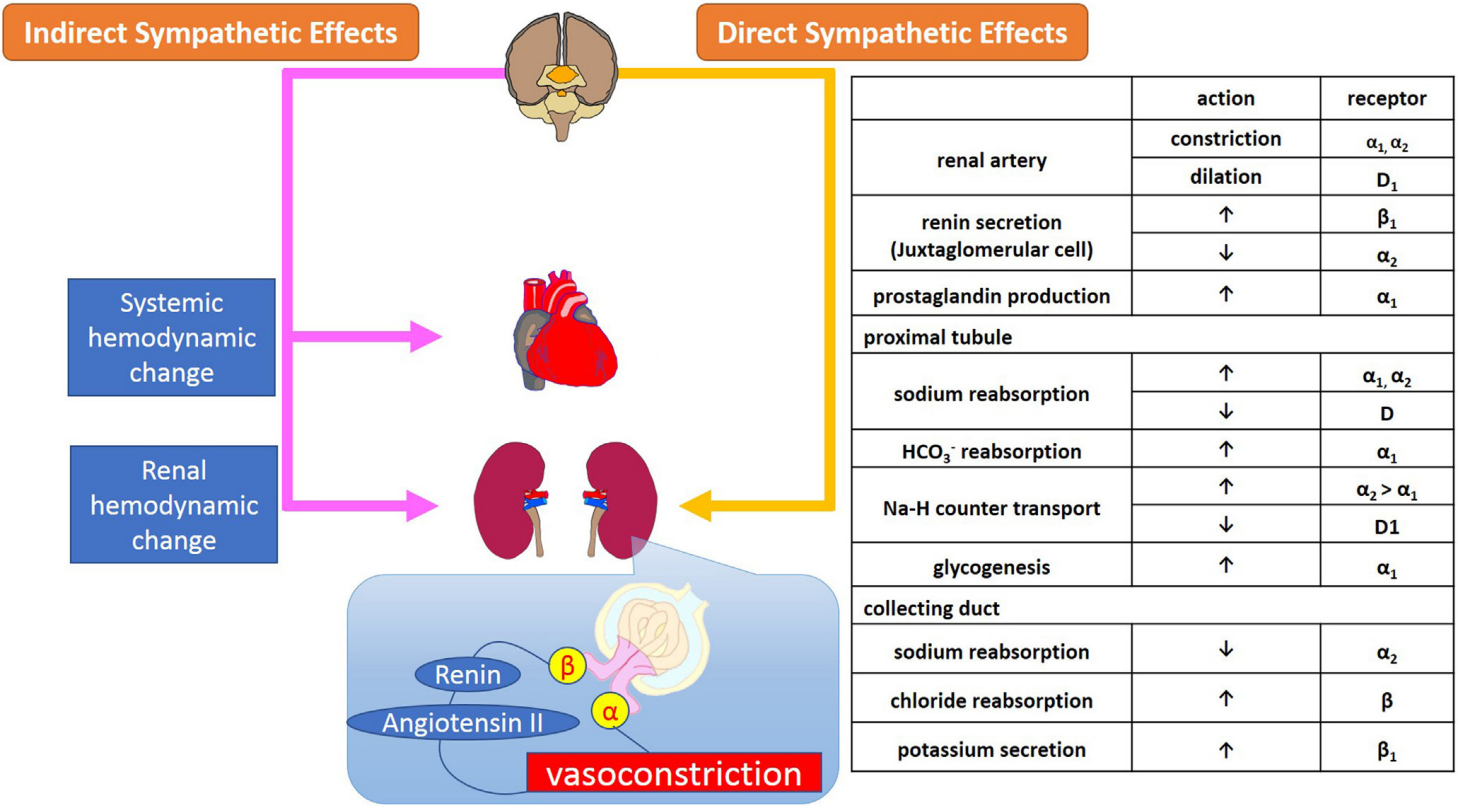
Sympathetic regulation of renal function; glomerular filtration and reabsorption of electrolytes, sodium, and water in kidneys. Sympathetic regulation of kidney function consists of systemic and renal hemodynamic modulation, sympathetic effects *via* secretion of neurohumoral agents, and direct adrenergic and dopaminergic effects on receptors on renal arterioles and tubules.

The renal efferent nerves are predominantly adrenergic. Norepinephrine release mediates vasoconstriction of the renal vasculature, as well as sodium and water reabsorption at renal tubular epithelial cells, and renin release from the juxtaglomerular cells (15). Increased RSNA mediates contraction of smooth muscle cells of the arterial resistance vessels, which primarily leads to constriction of the afferent and efferent renal arterioles and subsequently, albeit to a smaller extent, of the interlobular arteries, resulting in a decreased renal blood flow. Sympathetic regulation of GFR, electrolyte levels, and sodium and water reabsorption in the kidneys is summarized in Figure 1.

The α_1_-adrenoreceptors are located on the renal vasculature, nephrons, and proximal tubules where they contribute to vasoconstriction, sodium reabsorption, glycogenesis, and production of prostaglandins (16). In the nephron, among the several subtypes of adrenoreceptors, α_1A_-receptors are primarily responsible for regulating renal blood flow and sodium and water reabsorption by the proximal tubules. The distribution of α_2_-adrenoreceptors in the kidneys is similar to that of α_1_-adrenoreceptors, however, while α_1_- and α_2_-adrenoreceptors have synergetic effects on sodium reabsorption at the level of the proximal tubules, α_2_-receptors also mediate diuresis at the collecting duct *via* suppression of secretion as well as inhibition of antidiuretic hormone (17).

Renal β_1_-adrenoreceptors are located on the juxtaglomerular cells, nephrons, distal tubules, and collecting ducts where they stimulate renin secretion and suppress potassium secretion. The β_2_-adrenoreceptors exist mainly in the proximal and distal tubules and collecting ducts. Stimulation of β-adrenoreceptors mediates reabsorption of Ca^+^ and Mg^+^ in the cortex, and of sodium chloride (NaCl) in the cortex and medulla through the activation of cAMP (18).

Dopamine D_1_-receptors are widely spread across the renal vasculature, proximal, and distal tubules, and collecting ducts and mediate vasodilatation and inhibition of sodium reabsorption *via* activation of adenylate cyclase (13). In contrast, D_2_-receptors are localized in nephrons and presynaptic sympathetic nerve terminals where they inhibit norepinephrine release through the suppression of adenylate cyclase (14).

## Central Mechanisms Regulating RSNA

**Figure 2:**
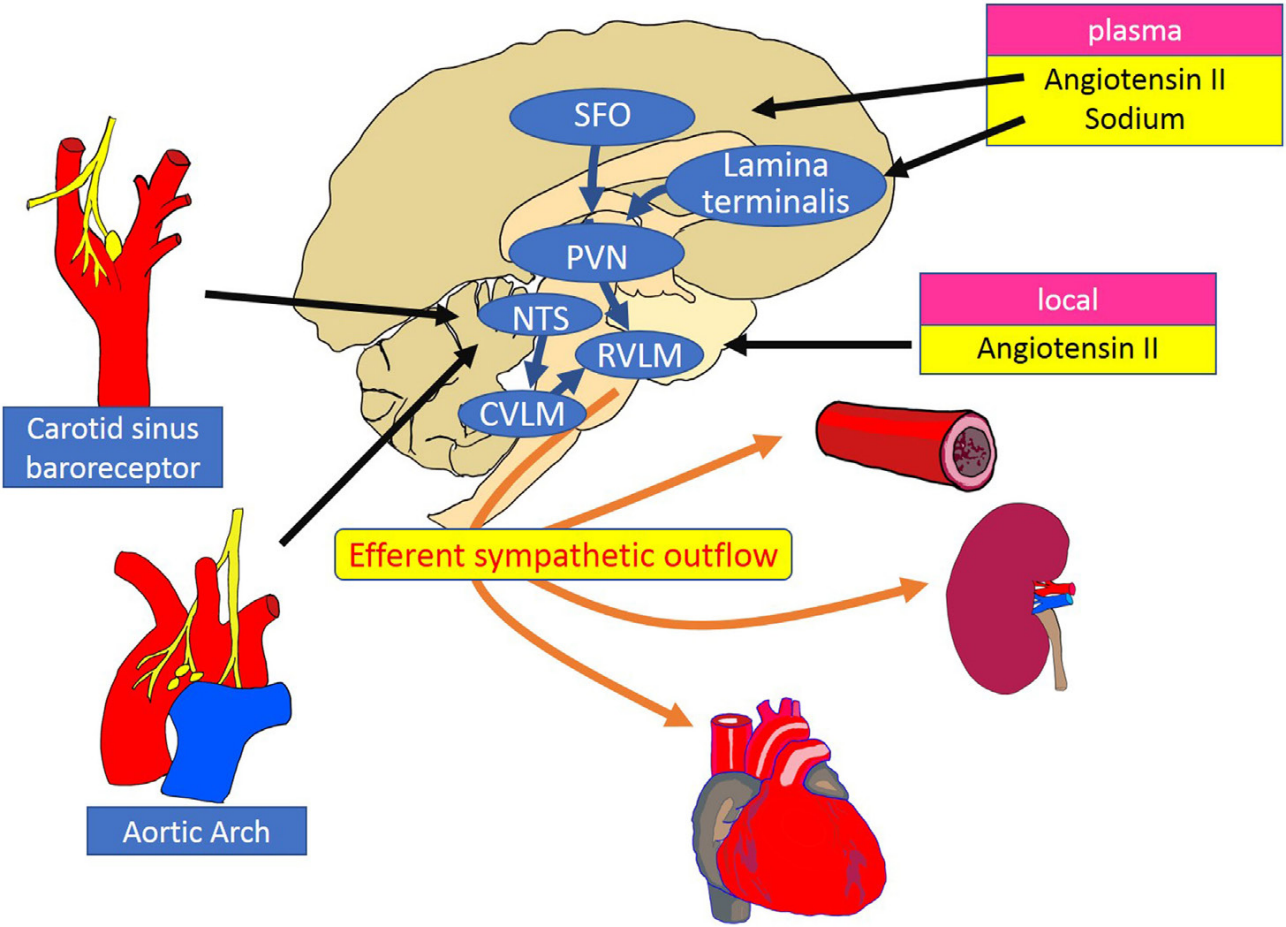
Schematic diagram of the central and peripheral mechanisms of sympathetic regulation of the heart, vessels, and kidneys. The RVLM plays a key role as a cardiovascular centre that receives and integrates peripheral signals providing information on blood pressure, fluid volume, and oxygen saturation. Instant changes in blood pressure are perceived by baroreceptors and transferred to the NTS as an input signal of baroreflex control of sympathetic outflow. Stimulation of the SFO by circulating angiotensin II increases the efferent sympathetic activity through the activation of the PVN and the RVLM neurons. Inhibitory pathways are activated between the lamina terminalis and PVN in response to plasma sodium. Increased activity of the RVLM neurons is transmitted to the intermediolateral cell column of the spinal cord, where peripheral sympathetic nerves to the heart, arteries and kidneys are activated. RVLM, rostral ventrolateral medulla; NTS, nucleus tractus solitarius; CVLM, caudal ventrolateral medulla; PVN, paraventricular nucleus; SFO, subfornical organ.

The level of RSNA is dependent on the neuronal activity in sympathetic premotor nuclei in the brainstem and hypothalamus, including the rostral ventrolateral and ventromedial medulla [rostral ventrolateral medulla (RVLM), RVMM] and the paraventricular nucleus (PVN). The RVLM is sympatho-excitatory and plays a pivotal role in the regulation of efferent renal nerve activity. Neurons in the RVLM project to pre-ganglionic neurons in the intermediolateral cell column of the spinal cord, which *via* postganglionic neurons, project to peripheral organs such as heart, arteries, and kidneys (19). The remarkable reduction in blood pressure after destruction of premotor neurons in the RVLM is evidence of its important role (20). The activity of premotor neurons in the RVLM and PVN is modulated by renal mechano and chemoreceptor reflexes mediated *via* renal afferent nerves (4). Central and peripheral mechanisms of sympathetic regulation of the kidney are summarized in Figure 2 (19).

In response to an increase in blood pressure, activation of the carotid sinus and aortic depressor nerves stimulates neurons in the nucleus tractus solitarius (NTS), which project and activate neurons in the caudal ventrolateral medulla (CVLM). The neurotransmission between CVLM and RVLM is mediated by inhibitory GABAergic neurons, which suppresses neuronal activity in the RVLM, reduces sympathetic nerve activity, and thus decreases blood pressure (19). Renal afferent sensory nerves project to the RVLM *via* NTS and PVN, where there is integration of afferent signals from the kidney, elicited by events such as ischemia, oxidative stress, and altered angiotensin II and glucose levels. The importance of renal afferent reflexes has been demonstrated by the finding that the increase in norepinephrine secretion from the hypothalamus induced by kidney injury (21) was abolished by afferent renal denervation in rats (22).

In the brain, there are numerous neurotransmitters that modulate sympathetic nerve activity, one of these is nitric oxide (NO) that acts as both a neurotransmitter and a neuromodulator (23). Endogenous NO production, induced by neuronal NO synthase (NOS) and inducible NOS, appear to have different effects on blood pressure and sympathetic nervous system activity (24, 25). This was considered at least partly attributable to the different amount of neurotransmitter released; namely sympatho-excitatory l-glutamate and inhibitory GABA within the RVLM (25). Microinjection of exogenous NO suggests cyclic 3′-5′ guanosine monophosphate-dependent mechanisms in the modulation of neuronal activity (26).

The effects of NO system activation within the central sympathetic nervous system are also mediated by the suppression of angiotensin II release. Since central angiotensin II is elevated and stimulates superoxide radical generation in cardiovascular diseases, the NO-mediated modulation of sympathetic nervous system is severely impaired in subjects with hypertension or end stage renal failure (25, 27). In Wistar Kyoto rats (WKY), overexpression of inducible NOS in the RVLM reportedly increased blood pressure, which was associated with sympathetic overactivity, and was attenuated by the antioxidant tempol (24). Inhibition of neuronal oxidative stress may, therefore, represent an effective approach to reduce neurohumoral activation in cardiovascular diseases and renal failure.

## Afferent Sensory Renal Nerves

The majority of renal afferent nerves originate in the proximal ureter, around large vessels, and in the adventitia and the smooth muscle layer of the renal pelvis. In the renal pelvis, renal efferent and afferent nerve fibers distribute separately despite them being intertwined in the same nerve bundle (28). Circumferential distribution of the afferent nerve fibers results in an ideal stretch receptor covering the renal pelvic wall. Renal afferent nerves are also chemo-sensitive as shown by the finding that ischemic metabolites and uremic toxins evoke renal efferent nerve activation through a reflex arc in animals and patients with end-stage renal failure (29).

The neural cell bodies of renal afferent nerves are typically located in the dorsal root ganglia predominantly T12-L1 in rats and monkeys (4). Immunohistochemical studies revealed calcitonin gene-related peptide (CGRP) as a marker of renal afferent nerves (4). CGRP is also highly concentrated in the renal medulla, papilla, and cortex. With retrograde tracing techniques, more than 90% of dorsal root ganglia cells were found CGRP positive in rats, with a marked decrease after neonatal treatment with capsaicin (30).

Afferent RSNA is modulated by efferent renal sympathetic nerves *via* norepinephrine as the neurotransmitter. Efferent renal sympathetic nerves activate α_1_- and α_2_-adrenoreceptors on afferent renal nerves, which increases and decreases afferent renal nerve activity, respectively (31). Such interactions are essential in blood pressure regulation, particularly in the context of a high-salt diet. The reduction in RSNA in response to a high salt intake decreases urinary sodium retention to prevent further increase in blood pressure. There is also evidence for a direct central inhibitory action of sodium on RSNA (32, 33). Dietary modulation of efferent RSNA is, therefore, a key mechanism of maintenance of volume, sodium, and BP homeostasis (31).

## Renorenal Reflexes

The renal afferent nerves play a key role within the reflex arc controlling RSNA. The stimuli received at peripheral receptors are transferred *via* afferent inputs to central regulatory structures, which in turn generate efferent signals targeted at various peripheral organs (34).

**Figure 3:**
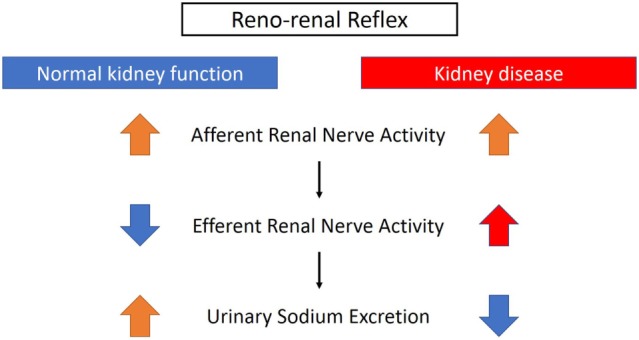
Different effects of renorenal reflexes with normal or impaired kidney function. With normal kidney function, the renorenal reflex operates as an inhibitory response, in which activation of renal sensory nerves by stimuli, including increased renal pelvic pressure, renal venous pressure, and renal pelvic administration of substance P, bradykinin, or capsaicin, decreases efferent renal sympathetic nerve activity that in turn increases urinary sodium excretion. When renal function is impaired, for example, with ischemic kidneys, the inhibitory reflex is attenuated and activation of afferent renal nerves results in a sympathoexcitatory reflex.

The renorenal reflex is an inhibitory reflex where stretch activation of renal afferent nerves elicits a decrease in ipsilateral and contralateral efferent renal nerve activity to exhibit compensatory natriuresis and diuresis (Figure 3) (15). In a study of anesthetized rats with volume overload, total unilateral renal denervation (efferent + afferent denervation) resulted in an increase in contralateral efferent RSNA associated with decrease in contralateral sodium excretion (35). These results indicate that efferent renal nerve activity, which was suppressed by a volume-induced increase in contralateral renal afferent activity, was increased by contralateral renal denervation. Intriguingly, although unilateral renal denervation induced ipsilateral diuresis and natriuresis, total sodium excretion and urine volume from both kidneys remained unchanged, providing functional evidence for the afferent renal nerve exerting a tonic inhibitory reflex on both ipsilateral and contralateral efferent renal nerve activity. The integrated system of renorenal reflex contributes to homeostatic regulation of sodium–water balance and blood pressure.

On the other hand, as discussed above, renal efferent and afferent nerves also operate *via* a negative feedback system where an increase in renal efferent nerve activity elevates renal afferent activity, which in turn suppresses renal efferent activity. This negative feedback operation also contributes to homeostasis, by preventing overactivation of renal sympathetic nerves and subsequent excessive sodium retention (15). However, in the condition of renal disease, this negative feedback system is attenuated and there is overactivation of renal afferent nerve activity that does not suppress renal efferent nerve activity, but leads to sympathoexcitation, vasoconstriction, and subsequently an increase in blood pressure (15) (Figure 3).

## Activation of the Sympathetic Nervous System in Hypertension and Kidney Disease

There is solid evidence for sympathetic nerve overactivity in the development of hypertension as demonstrated by: (i) muscle sympathetic nerve activity (MSNA) being predominantly increased in early human hypertension (36), (ii) renal norepinephrine spillover being substantially elevated in essential hypertension and most other forms of hypertension including renal hypertension (both renovascular as well as renoparenchymal) (37), and (iii) renal sympathetic nerve firing being doubled in Spontaneously Hypertensive Rat (SHR) compared with WKY (Wistar Kyoto Rat) (38), suggesting that renal sympathetic nerve overactivity is a common pathway for the development of hypertension.

Strong links have also been demonstrated between elevated blood pressure and increased sympathetic nerve activity in various stages of chronic kidney disease. Campese et al. demonstrated that increased stimulation of renal sensory nerves is an important factor in the development of treatment-resistant hypertension, likely more relevant than renin–angiotensin system activation or volume overload (39). Chemical substances such as adenosine and bradykinin are considered responsible for stimulation of parenchymal chemoreceptors in diseased and ischemic kidneys.

The important role of afferent renal nerves on overall sympathetic nervous system activity in kidney disease is well documented in patients with end-stage renal failure and post-renal transplant. The increased afferent renal nerve activity arising from the diseased kidneys, which mediates increases in systemic sympathetic nerve activity and hypertension, was sustained in patients with end-stage renal failure after the initiation of hemodialysis or even after transplantation of a donor kidney if both native kidneys remained *in situ* (29). However, blood pressure and MSNA were normalized after bilateral nephrectomy, indicating that the diseased kidneys are the origin of stimuli, through afferent signaling, which enhance sympathetic nerve activity.

## Renal Denervation as a Neuromodulatory Approach for Hypertension and CKD

Based on the evidence presented above, targeting the renal sympathetic nerves is a logical therapeutic strategy for hypertension and kidney disease. In fact, the initial evidence of benefits of renal denervation was demonstrated by transplantation of the diseased kidney in patients with end-stage renal failure, which improved blood pressure control (6). Subsequently, several attempts at eliminating sympathetic innervation of the kidneys in humans were demonstrated by surgical sectioning of thoracic and lumber nerves and splanchnicectomy, which successfully reduced blood pressure and improved prognosis, but was associated with significant side effects (40). Further animal studies also revealed the beneficial effects of systemic sympathoinhibition on natriuresis and improvement of cardiac function (41–43). The effects of renal denervation on renal blood flow are controversial. Renal blood flow was reportedly unchanged between denervated and innervated kidneys after unilateral renal denervation (44), and after total denervation (45), suggesting that sympathetic effects on renal blood flow are marginal under healthy conditions. On the other hand, Osborn et al. found decrease in renal blood flow and blood pressure in rats (46).

## Renal Denervation—Symplicity Trials

The Symplicity HTN-1 trial provided the first-in-man study aimed at achieving renal sympathetic denervation using a catheter-based technique. In this proof-of-concept study, a significant reduction in office systolic/diastolic blood pressure in patients with treatment-resistant hypertension was demonstrated up to 12 months post-denervation (−14/−10, −21/−10, −22/−11, −24/−11, and −27/−17 mmHg at 1, 3, 6, 9, and 12 months after the procedure, respectively) (2). The subsequent HTN-2 trial was a randomized controlled study that compared the effects of blood pressure reduction between a renal denervation group and conventional treatment group. Consistent with HTN-1, renal denervation demonstrated a significant reduction of office systolic blood pressure (by −32 ± 23/12 ± 11 mmHg at 6 months, and by −28 ± 25/10 ± 11 mmHg at 12 months follow-up), whereas pharmacological treatment alone did not further decrease blood pressure (1 ± 21/0 ± 10 mmHg) (47, 48).

The change in ambulatory systolic blood pressure was consistent with that of office reading but less pronounced (HTN-1, −6 vs −27 mmHg; HTN-2, −11 ± 7 vs −32 ± 23 mmHg) (2, 47). The discrepancy between ambulatory and office blood pressure reduction after renal denervation was larger than other unblinded drug trials (49), which has raised some criticism in that blood pressure reduction in the renal denervation group might be influenced by regression to the mean effect. The unchanged blood pressure of the control group in HTN-2, however, might explain the exact effects of renal denervation on blood pressure. Nonetheless, potential information bias remained since the control groups were not blinded.

Further studies revealed long-term effects of catheter-based renal denervation on blood pressure (−32/−14 mmHg at 36 months) (50), a significant and sustained reduction in sympathetic nerve activity (1, 43), and comparable beneficial effects for patients with chronic kidney disease despite some rare complications (51–53). Accordingly, renal denervation also appeared to be a logical treatment option for cardiorenal diseases where renal afferent signaling from the damaged kidney to the brain evokes systemic activation of the sympathetic nervous system. In contrast to the results of Symplicity HTN-1 and 2, the Symplicity HTN-3 study, a large, randomized, blinded, sham-controlled study, demonstrated marked reductions in office blood pressure at 6 months after the procedure, but this change was not statistically significant compared with that in the sham-control group (−14.1 ± 23.9 vs −11.7 ± 25.9 mmHg, *p* = 0.26) (54). Critical review of the study revealed that the office blood pressure reduction of the denervation group was less pronounced when compared with previous Symplicity trials (−14.1 ± 23.9/−6.6 ± 11.9 in HTN-3 vs −22/−11 mmHg in HTN-1 and −32 ± 23/−12 ± 11 mmHg in HTN-2 at 6 months follow-up) (47, 48, 50, 54). Procedural shortcomings, including not achieving a circumferential ablation pattern, operator inexperience, and insufficient proctoring were discussed as possible contributors to this scenario. Indeed, renal denervation procedures in Symplicity HTN-3 study were performed by a total of 111 operators throughout the United States. Among them, 31% (34 operators) only performed 1 procedure, and 77% (85 operators) performed less than five procedures (54, 55). In the meantime, analyses of anatomical distribution of peri-arterial renal sympathetic nerves provided important information that emphasizes the need for optimization of the procedure to achieve more effective and consistent denervation (56, 57).

The most recent SPYRAL HTN-OFF MED trial (58) used a newly designed multielectrode radiofrequency Spyral catheter and applied a more aggressive treatment approach including denervation in the renal artery branches. The change in 24 h ambulatory blood pressure was compared between the renal denervation and sham procedure groups at 3 months in patients with mild-to-moderate hypertension (office systolic blood pressure >140 mmHg). Patients were drug-naive or taken off their antihypertensive medications, thereby avoiding potential bias through non-adherence with prescribed medication (59). Analysis of data from 80 patients (*n* = 38 in the denervation group vs *n* = 42 in the sham-control group) showed that the 3-month change in 24 h ambulatory and office blood pressures from baseline were significantly greater in the renal denervation than in the sham denervation group: 24 h ambulatory systolic/diastolic blood pressure −5.0/−4.4 mm Hg (95% CI −9.9 to −0.2; *p* = 0.0414 for systolic, and −7.2 to −1.6; *p* = 0.0024 for diastolic), office systolic/diastolic blood pressure −7.7/−4.9 mm Hg (−14.0 to −1.5; *p* = 0.0155 for systolic, and −8.5 to −1.4; *p* = 0.0077 for diastolic) (58). A summary of the Symplicity trials is shown in Table 1.

**Table 1:** Summary and demographics of Symplicity trials.

	HTN-1	HTN-2		HTN-3		SPYRAL HTN-OFF MED			
No. of patients	**RDN**	**RDN**	**Control**	**RDN**	**Control**	**RDN**	**Control**
	45	52	54	364	171	38	42
Catheter	Flex					Spyral multielectrode	
No. of ablation	up to 12	NR		11.2		43 (main + branch)	
No. of antihypertensive medications at baseline	4.7 ± 1.4	5.2 ± 1.5	5.3 ± 1.8	5.1 ± 1.4	5.2 ± 1.4	0	0
Aldosterone antagonist at baseline (% of patients)	NR	17	17	22.5	28.7	0.0	0.0
Office systolic blood pressure at baseline (mm Hg)	177 ± 20	178 ± 18	178 ± 16	179.7 ± 16.1	180.2 ± 16.8	162 ± 7.6	161.4 ± 6.4
Heart rate at baseline (beats/min)	72 ± 11	75 ± 15	71 ± 15	NR	NR	71.1 ± 11.0	73.4 ± 9.8
Change in office systolic blood pressure at 6 mo (mm Hg)						at 3 mo (mmHg)	

Absolute change	−22	−32 ± 23	1 ± 21	−14.1 ± 23.9	−11.7 ± 25.9	−10	−2.3
Change relative to control group	–	−33		−2.4		−7.7	
Change in 24-h ambulatory systolic blood pressure at 6 mo (mm Hg)						at 3 mo (mmHg)	

Absolute change	–	−11 ± 15	−3 ± 19	−6.8 ± 15.1	−4.8 ± 17.2	−5.5	−0.5
Change relative to control group	–	−8		−1.96		−5	
Change in antihypertensive medication (% of patients)							

Decrease in dose or no. of medications	22	20	6	NR	NR	0	0
Increase in dose or no. of medications	10	8	12	NR	NR	0	0

Despite these positive results, caution is still needed in the interpretation of the decrease in blood pressure observed in this trial. There was a large intra-group (between patients) variation in the change of blood pressure after denervation, indicating that the size of the decrease in blood pressure is heterogeneous despite the large number of ablations (average 43 ablations each patient) with a circumferential pattern. A possible explanation is anatomical variability in the course of renal innervation, which are rather common in animal experiments. Further and longer term studies are required to confirm these promising results and to optimize the treatment algorithm. Similarly, continued efforts to explore means to confirm the degree of renal denervation achieved will be important.

## Renal Denervation—Issues to be Solved

There remain a few issues to be solved regarding renal denervation.

First the lack of a simple, reliable, and reproducible test to measure sympathetic nervous activity in humans hampers the quantitative assessment of the degree of renal denervation, which represents an ongoing issue. Currently, in contrast to animal experiments, a reliable test to confirm that successful renal denervation has actually been achieved is limited to a combination of MSNA and invasive renal norepinephrine spillover methodology, neither of which are widely available. In addition, a consistent predictive marker of responders to renal denervation is yet to be identified. We have recently reported that ambulatory arterial stiffness index (AASI), a novel, clinically validated, and yet simple index of arterial stiffness appeared useful in the prediction of the blood pressure response to renal denervation (60). In patients with resistant hypertension, high AASI (stiff artery) was associated with low MSNA and had a poor blood pressure response to renal denervation. Whereas those with low AASI (compliant artery) was associated with high MSNA and demonstrated remarkable blood pressure response as well as reduction in MSNA after renal denervation, suggesting neural contribution to their hypertension. These results suggest that neurogenic hypertension is most suitable for renal denervation and although the reduction in MSNA and norepinephrine spillover might not always correlate with blood pressure lowering, sympathoinhibition is an essential effect of renal denervation.

Carotid sinus baroreflex is an essential and powerful regulatory system of blood pressure and is known to “reset” to a higher blood pressure range in hypertension (3). Electrical stimulation of carotid sinus baroreceptors has been shown to be effective in both animal (61) and human (62) studies, demonstrating a significant reduction of blood pressure and suppression of sympathetic nervous activity in hypertension. In addition to the strong sympathomodulatory effects, the carotid sinus stimulation has some advantages; the stimulation protocol can be individually adjusted and can be even switched off in case of hemodynamic instability, which is not feasible with renal denervation. Despite that, a significant downside is the invasive nature of device implantation and related complications such as infection, damage of the arteries and nerves, and stroke (63). Lohmeier et al. have extensively explored the physiological effects of baroreflex activation from the view of renal sympathetic nerves. The sympathoinhibitory and hypotensive effects of baroreceptor activation are independent from the presence of renal nerves, suggesting that renal denervation and baroreflex activation might have different mechanisms of action (64). Yet, there have been an ongoing controversy over which is a superior device-based therapy for hypertension, renal denervation, or carotid sinus stimulation (65, 66).

## Where to Next: Selective Renal Deafferentation for Chronic Kidney Disease?

Given the key role of afferent renal nerves in kidney disease, experimental evidence supports the potential therapeutic utility of deafferentation in chronic kidney disease. Selective renal afferent denervation by dorsal rhizotomy prevented the progression of kidney disease and abolished the increased blood pressure and norepinephrine turnover induced by 5/6th nephrectomy in experimental kidney disease models (22).

As mentioned above, afferent renal nerves are also important in the response of the renorenal reflex to a high salt diet, which modulates efferent RSNA and urinary sodium and water excretion. Rats fed a high-salt diet increase urinary sodium excretion to maintain blood pressure at normal levels, whereas rats fed a high-salt diet and with selective deafferentation (dorsal rhizotomy) increase urinary sodium excretion only by increasing blood pressure by 30 mmHg (67). This suggests that (1) renal afferents are essential in the adaptive control of urinary sodium excretion when fed a high-salt diet, and (2) renal deafferentation might shift the pressure-natriuresis curve toward a higher pressure range, and elicit susceptibility to the hypertensive effects of high-salt diet. However, further studies are necessary to address whether in subjects with kidney disease renal deafferentation benefits blood pressure control by suppression of efferent RSNA or worsens blood pressure control by conferring salt-sensitive hypertension.

A possible future application of deafferentation would be to target patients with heart failure and concomitant renal disease (cardiorenal syndrome), who tend to have a narrow window of fluid volume control, which requires large doses of diuretics. The accompanying kidney impairment often makes heart failure difficult to control due to the limited sensitivity and tolerability to diuretics. In heart failure, renal deafferentation may potentially reduce renal sympathoexcitation, which commonly leads to excessive sodium reabsorption and hypertension in patients with the cardiorenal syndrome. However, taking the enhanced salt-sensitivity and altered pressure-natriuresis relationship into consideration, deafferentation technique needs to be carefully applied to patients with cardiorenal syndrome, and restriction of salt intake is warranted.

## Reinnervation of Renal Nerves

Whether or not regeneration of renal nerves affects the long-term responses to renal denervation is controversial. Given that autotransplanted kidneys, which are without renal nerves in the early stage of posttransplantation (7), function normally and respond to diuretics suggests that the renal nerves are not essential for renal functional capacity (4). Some findings suggest that that reinnervation of renal nerves may begin as early as 28 days in human (68). Similar phenomena were observed in dogs, where renal autografts were reinnervated within 3–6 months after transplantation (69). In a recent study in normotensive sheep, the time-dependent nature of functional renal innervation was observed after catheter-based renal denervation (70). Transarterial ablation of renal nerves was performed using the Symplicity Flex Catheter, and arterial pressure, heart rate, renal blood flow, and renal vascular conductance were measured in response to electric stimulation of the renal nerve and compared before and after renal denervation. Although the response of RSNA (RSNA) to electric stimulation was abolished immediately after renal denervation, anatomical reinnervation of renal efferent and afferent nerves was observed at 5 and 11 months post-procedure, as indicated by the presence of tyrosine hydroxylase and CGRP staining. Functional re-innervation was also documented by the presence of RSNA and the return of the response to nerve stimulation at 11 months. These results indicated that there may be a time-dependent effect of renal denervation on renal innervation. It remains to be established if reinnervation of the renal nerves occurs in the presence of hypertension or heart failure.

## Conclusion

Renal efferent and afferent nerves play a major role in the control of renal and cardiovascular homeostasis. Information from the renal parenchyma is relayed *via* the afferent renal nerves to central autonomic nuclei, where the information is integrated with inputs from other neural reflexes to determine the level of sympathetic outflow to individual organs. In particular, the renorenal reflex plays an important role in determining the level of RSNA and renal function.

The renal sympathetic nerves play a major role in the regulation of renal function and fluid homeostasis in the normal, healthy state. In situations where kidney function deteriorates, or in the presence of conditions such as heart failure or hypertension, altered levels of RSNA contribute and worsen the abnormalities in renal and cardiovascular homeostasis.

The etiology of cardiovascular diseases is multifactorial, but is typically characterized by a substantial sympathetic contribution. While reasonable caution is needed, sympatho-modulatory approaches to target the renal nerves are likely to develop into important future treatment options for cardiovascular disease.

## Author Contributions

All authors contributed to drafting of the manuscript and critical review.

## Conflict of Interest Statement

MS was an investigator in studies sponsored by Medtronic. The laboratories of MS received research funding from Medtronic, Abbott, and Servier Australia. MS serves on scientific advisory boards for Abbott, BI, Novartis, and Medtronic and has received honoraria and travel support from Abbott, BI, Servier, Novartis, and Medtronic. The other authors declare no competing interests.

## References

[1] Schlaich MP Sobotka PA Krum H Lambert E Esler MD Renal sympathetic-nerve ablation for uncontrolled hypertension. N Engl J Med (2009) 361:932–4. 10.1056/NEJMc0904179 19710497

[2] Krum H Schlaich M Whitbourn R Sobotka PA Sadowski J Bartus K Catheter-based renal sympathetic denervation for resistant hypertension: a multicentre safety and proof-of-principle cohort study. Lancet (2009) 373:1275–81. 10.1016/S0140-6736(09)60566-3 19332353

[3] Sata Y Kawada T Shimizu S Kamiya A Akiyama T Sugimachi M. Predominant role of neural arc in sympathetic baroreflex resetting of spontaneously hypertensive rats. Circ J (2015) 79:592–9. 10.1253/circj.CJ-14-1013 25746544

[4] DiBona GF Kopp UC Neural control of renal function. Physiol Rev (1997) 77:75–197. 10.1152/physrev.1997.77.1.75 9016301

[5] Bernard C Lecons sur les Proprietes Physiologiques et les Alterations pathologiques des liquids de l’organism. Paris: Balliere et Fils (1859) 2:170–1.

[6] Carrel A Guthrie CC Anastomosis of blood vessels by the patching method and transplantation of the kidney. J Am Med Assoc (1906) 47:1648–51. 10.1001/jama.1906.25210200044001h PMC2588785 11697483

[7] Quinby WC The action of diruetics on the denervated kidney. Am J Physiol (1917) 42:593–4.

[8] Drukker J Groen GJ Boekelaar AB Baljet B. The extrinsic innervation of the rat kidney. Clin Exp Hypertens A (1987) 9(Suppl 1):15–31. 3677451 10.3109/10641968709160161

[9] Page IH The relationship of the extrinsic renal nerves to the origin of experimental hypertension. Am J Physiol (1935) 112:166–71. 10.1152/ajplegacy.1935.112.1.166

[10] Barajas L Liu L Powers K. Anatomy of the renal innervation: intrarenal aspects and ganglia of origin. Can J Physiol Pharmacol (1992) 70:735–49. 10.1139/y92-098 1423018

[11] McKenna OC Angelakos ET Adrenergic innervation of the canine kidney. Circ Res (1968) 22:345–54. 10.1161/01.RES.22.3.345 5644181

[12] Dibona GF. Renal innervation and denervation: lessons from renal transplantation reconsidered. Artif Organs (1987) 11:457–62. 10.1111/j.1525-1594.1987.tb02710.x 3326559

[13] Yamaguchi I Jose PA Mouradian MM Canessa LM Monsma FJ Jr Sibley DR Expression of dopamine D1A receptor gene in proximal tubule of rat kidneys. Am J Physiol (1993) 264:F280–5. 8447437 10.1152/ajprenal.1993.264.2.F280

[14] Arinami T Gao M Hamaguchi H Toru M. A functional polymorphism in the promoter region of the dopamine D2 receptor gene is associated with schizophrenia. Hum Mol Genet (1997) 6:577–82. 10.1093/hmg/6.4.577 9097961

[15] Johns EJ Kopp UC DiBona GF Neural control of renal function. Compr Physiol (2011) 1:731–67. 10.1002/cphy.c100043 23737201

[16] Morrow AL Creese I. Characterization of alpha 1-adrenergic receptor subtypes in rat brain: a reevaluation of [3H]WB4104 and [3H]prazosin binding. Mol Pharmacol (1986) 29:321–30. 3010073

[17] Pettinger WA Umemura S Smyth DD Jeffries WB. Renal alpha 2-adrenoceptors and the adenylate cyclase-cAMP system: biochemical and physiological interactions. Am J Physiol (1987) 252:F199–208. 3028168 10.1152/ajprenal.1987.252.2.F199

[18] Bailly C Imbert-Teboul M Roinel N Amiel C. Isoproterenol increases Ca, Mg, and NaCl reabsorption in mouse thick ascending limb. Am J Physiol Renal Physiol (1990) 258:F1224–31. 10.1152/ajprenal.1990.258.5.F1224 2337151

[19] Kumagai H Oshima N Matsuura T Iigaya K Imai M Onimaru H Importance of rostral ventrolateral medulla neurons in determining efferent sympathetic nerve activity and blood pressure. Hypertens Res (2012) 35:132–41. 10.1038/hr.2011.208 22170390 PMC3273996

[20] Guertzenstein PG Silver A. Fall in blood pressure produced from discrete regions of the ventral surface of the medulla by glycine and lesions. J Physiol (1974) 242:489–503. 10.1113/jphysiol.1974.sp010719 4455831 PMC1330679

[21] Ye S Ozgur B Campese VM. Renal afferent impulses, the posterior hypothalamus, and hypertension in rats with chronic renal failure. Kidney Int (1997) 51:722–7. 10.1038/ki.1997.103 9067904

[22] Campese VM Kogosov E. Renal afferent denervation prevents hypertension in rats with chronic renal failure. Hypertension (1995) 25:878–82. 10.1161/01.HYP.25.4.878 7721447

[23] Garthwaite J Boulton CL Nitric oxide signaling in the central nervous system. Annu Rev Physiol (1995) 57:683–706. 10.1146/annurev.ph.57.030195.003343 7539993

[24] Kimura Y Hirooka Y Sagara Y Ito K Kishi T Shimokawa H Overexpression of inducible nitric oxide synthase in rostral ventrolateral medulla causes hypertension and sympathoexcitation via an increase in oxidative stress. Circ Res (2005) 96:252–60. 10.1161/01.RES.0000152965.75127.9d 15591232

[25] Zanzinger J. Mechanisms of action of nitric oxide in the brain stem: role of oxidative stress. Auton Neurosci (2002) 98:24–7. 10.1016/S1566-0702(02)00025-5 12144034

[26] Schwarz P Diem R Dun NJ Forstermann U. Endogenous and exogenous nitric oxide inhibits norepinephrine release from rat heart sympathetic nerves. Circ Res (1995) 77:841–8. 10.1161/01.RES.77.4.841 7554131

[27] Leone A Moncada S Vallance P Calver A Collier J. Accumulation of an endogenous inhibitor of nitric oxide synthesis in chronic renal failure. Lancet (1992) 339:572–5. 10.1016/0140-6736(92)90865-Z 1347093

[28] Mulder J Hokfelt T Knuepfer MM Kopp UC Renal sensory and sympathetic nerves reinnervate the kidney in a similar time dependent fashion following renal denervation in rats. Am J Physiol Regul Integr Comp Physiol (2013) 304:R675–82. 10.1152/ajpregu.00599.2012 23408032 PMC3627950

[29] Converse RL Jr Jacobsen TN Toto RD Jost CM Cosentino F Fouad-Tarazi F Sympathetic overactivity in patients with chronic renal failure. N Engl J Med (1992) 327:1912–8. 10.1056/NEJM199212313272704 1454086

[30] Su HC Wharton J Polak JM Mulderry PK Ghatei MA Gibson SJ Calcitonin gene-related peptide immunoreactivity in afferent neurons supplying the urinary tract: combined retrograde tracing and immunohistochemistry. Neuroscience (1986) 18:727–47. 10.1016/0306-4522(86)90066-7 2427972

[31] Kopp UC Cicha MZ Smith LA Ruohonen S Scheinin M Fritz N Dietary sodium modulates the interaction between efferent and afferent renal nerve activity by altering activation of alpha2-adrenoceptors on renal sensory nerves. Am J Physiol Regul Integr Comp Physiol (2011) 300:R298–310. 10.1152/ajpregu.00469.2010 21106912 PMC3043795

[32] Frithiof R Xing T McKinley MJ May CN Ramchandra R. Intracarotid hypertonic sodium chloride differentially modulates sympathetic nerve activity to the heart and kidney. Am J Physiol Regul Integr Comp Physiol (2014) 306:R567–75. 10.1152/ajpregu.00460.2013 24523342 PMC4043129

[33] May CN McAllen RM. Brain angiotensinergic pathways mediate renal nerve inhibition by central hypertonic NaCl in conscious sheep. Am J Physiol Regul Integr Comp Physiol (1997) 272:R593–600. 10.1152/ajpregu.1997.272.2.R593 9124483

[34] DiBona GF. Neural control of the kidney: functionally specific renal sympathetic nerve fibers. Am J Physiol Regul Integr Comp Physiol (2000) 279:R1517–24. 10.1152/ajpregu.2000.279.5.R1517 11049831

[35] Colindres RE Spielman WS Moss NG Harrington WW Gottschalk CW. Functional evidence for renorenal reflexes in the rat. Am J Physiol (1980) 239:F265–70. 7435566 10.1152/ajprenal.1980.239.3.F265

[36] Grassi G Colombo M Seravalle G Spaziani D Mancia G. Dissociation between muscle and skin sympathetic nerve activity in essential hypertension, obesity, and congestive heart failure. Hypertension (1998) 31:64–7. 10.1161/01.HYP.31.1.64 9449392

[37] Esler M Lambert G Rocca BL Vaddadi G Kaye D. Sympathetic nerve activity and neurotransmitter release in humans: translation from pathophysiology into clinical practice. Acta Physiol (2003) 177:275–84. 10.1046/j.1365-201X.2003.01089.x 12608997

[38] Lundin S RICKSTEN SE Thoren P. Renal sympathetic activity in spontaneously hypertensive rats and normotensive controls, as studied by three different methods. Acta Physiol (1984) 120:265–72. 10.1111/j.1748-1716.1984.tb00133.x 6711341

[39] Campese V Mitra N Sandee D. Hypertension in renal parenchymal disease: why is it so resistant to treatment? Kidney Int (2006) 69:967–73. 10.1038/sj.ki.5000177 16528245

[40] Smithwick RH Thompson JE Splanchnicectomy for essential hypertension; results in 1,266 cases. J Am Med Assoc (1953) 152:1501–4. 10.1001/jama.1953.03690160001001 13061307

[41] Jackson N Gizurarson S Azam MA King B Ramadeen A Zamiri N Effects of renal artery denervation on ventricular arrhythmias in a postinfarct model. Circ Cardiovasc Interv (2017) 10:e004172. 10.1161/CIRCINTERVENTIONS.116.004172 28258128

[42] McLellan AJ Schlaich MP Taylor AJ Prabhu S Hering D Hammond L Reverse cardiac remodeling after renal denervation: atrial electrophysiologic and structural changes associated with blood pressure lowering. Heart Rhythm (2015) 12:982–90. 10.1016/j.hrthm.2015.01.039 25638699

[43] Hering D Marusic P Walton AS Lambert EA Krum H Narkiewicz K Sustained sympathetic and blood pressure reduction 1 year after renal denervation in patients with resistant hypertension. Hypertension (2014) 64(1):118–24. 10.1161/HYPERTENSIONAHA.113.03098 24732891

[44] Barrett CJ Navakatikyan MA Malpas SC. Long-term control of renal blood flow: what is the role of the renal nerves? Am J Physiol Regul Integr Comp Physiol (2001) 280:R1534–45. 10.1152/ajpregu.2001.280.5.R1534 11294779

[45] Calzavacca P Bailey M Velkoska E Burrell LM Ramchandra R Bellomo R Effects of renal denervation on regional hemodynamics and kidney function in experimental hyperdynamic sepsis. Crit Care Med (2014) 42:e401–9. 10.1097/CCM.0000000000000302 24670939

[46] Jacob F Labine BG Ariza P Katz SA Osborn JW Renal denervation causes chronic hypotension in rats: role of β_1_-adrenergic activity. Clin Exp Pharmacol Physiol (2005) 32:255–62. 10.1111/j.1440-1681.2005.04179.x 15810988

[47] Esler MD Krum H Sobotka PA Schlaich MP Schmieder RE Bohm M. Renal sympathetic denervation in patients with treatment-resistant hypertension (The Symplicity HTN-2 Trial): a randomised controlled trial. Lancet (2010) 376:1903–9. 10.1016/S0140-6736(10)62039-9 21093036

[48] Esler MD Krum H Schlaich M Schmieder RE Bohm M Sobotka PA Renal sympathetic denervation for treatment of drug-resistant hypertension: one-year results from the Symplicity HTN-2 randomized, controlled trial. Circulation (2012) 126:2976–82. 10.1161/CIRCULATIONAHA.112.130880 23248063

[49] Howard JP Nowbar AN Francis DP. Size of blood pressure reduction from renal denervation: insights from meta-analysis of antihypertensive drug trials of 4,121 patients with focus on trial design: the CONVERGE report. Heart (2013) 99:1579–87. 10.1136/heartjnl-2013-304238 24038167

[50] Krum H Schlaich MP Sobotka PA Bohm M Mahfoud F Rocha-Singh K Percutaneous renal denervation in patients with treatment-resistant hypertension: final 3-year report of the Symplicity HTN-1 study. Lancet (2014) 383:622–9. 10.1016/S0140-6736(13)62192-3 24210779

[51] Hering D Mahfoud F Walton AS Krum H Lambert GW Lambert EA Renal denervation in moderate to severe CKD. J Am Soc Nephrol (2012) 23:1250–7. 10.1681/ASN.2011111062 22595301 PMC3380649

[52] Kiuchi MG Maia GL de Queiroz Carreira MA Kiuchi T Chen S Andrea BR Effects of renal denervation with a standard irrigated cardiac ablation catheter on blood pressure and renal function in patients with chronic kidney disease and resistant hypertension. Eur Heart J (2013) 34(28):2114–21. 10.1093/eurheartj/eht200 23786861

[53] Logan AG Diaconita V Ing DJ Oreopoulos GD Floras JS Rajan DK Acute renal failure after renal denervation. J Vasc Interv Radiol (2015) 26:450–1. 10.1016/j.jvir.2014.11.017 25735530

[54] Bhatt DL Kandzari DE O’Neill WW D’Agostino R Flack JM Katzen BT A controlled trial of renal denervation for resistant hypertension. N Engl J Med (2014) 370:1393–401. 10.1056/NEJMoa1402670 24678939

[55] Bhatt DL Future prospects for renal artery denervation. J Am Soc Hypertens (2016) 10(5):393–5. 10.1016/j.jash.2016.03.188 27067369

[56] Sakakura K Ladich E Cheng Q Otsuka F Yahagi K Fowler DR Anatomic assessment of sympathetic peri-arterial renal nerves in man. J Am Coll Cardiol (2014) 64:635–43. 10.1016/j.jacc.2014.03.059 25125292

[57] Hering D Marusic P Walton AS Duval J Lee R Sata Y Renal artery anatomy affects the blood pressure response to renal denervation in patients with resistant hypertension. Int J Cardiol (2016) 202:388–93. 10.1016/j.ijcard.2015.09.015 26432488

[58] Townsend RR Mahfoud F Kandzari DE Kario K Pocock S Weber MA Catheter-based renal denervation in patients with uncontrolled hypertension in the absence of antihypertensive medications (SPYRAL HTN-OFF MED): a randomised, sham-controlled, proof-of-concept trial. Lancet (2017) 390(10108):2160–70. 10.1016/S0140-6736(17)32281-X 28859944

[59] Kandzari DE Kario K Mahfoud F Cohen SA Pilcher G Pocock S The SPYRAL HTN Global Clinical Trial Program: rationale and design for studies of renal denervation in the absence (SPYRAL HTN OFF-MED) and presence (SPYRAL HTN ON-MED) of antihypertensive medications. Am Heart J (2016) 171:82–91. 10.1016/j.ahj.2015.08.021 26699604

[60] Sata Y Hering D Head G Walton A Peter K Marusic P Ambulatory arterial stiffness index as a predictor of blood pressure response to renal denervation. J Hypertens (2018). 10.1097/HJH.0000000000001682 29465712

[61] Lohmeier TE Irwin Rossing MA Serdar DJ Kieval RS. Prolonged activation of the baroreflex produces sustained hypotension. Hypertension (2004) 43:306–11. 10.1161/01.HYP.0000111837.73693.9b 14707159

[62] Heusser K Tank J Engeli S Diedrich A Menne J Eckert S Carotid baroreceptor stimulation, sympathetic activity, baroreflex function, and blood pressure in hypertensive patients. Hypertension (2010) 55:619–26. 10.1161/HYPERTENSIONAHA.109.140665 20101001

[63] Mayyas F Sturey T Van Wagoner DR Baroreflex stimulation versus renal denervation for treatment of hypertension: what constitutes a logical comparison of these interventions on atrial electrophysiology? J Cardiovasc Electrophysiol (2013) 24(9):1034–6. 10.1111/jce.12185 23773364 PMC3770820

[64] Lohmeier TE Hildebrandt DA Dwyer TM Barrett AM Irwin ED Rossing MA Renal denervation does not abolish sustained baroreflex-mediated reductions in arterial pressure. Hypertension (2007) 49:373–9. 10.1161/01.HYP.0000253507.56499.bb 17159083

[65] Schlaich MP Sata Y Esler MD CrossTalk opposing view: which technique for controlling resistant hypertension? Renal nerve ablation. J Physiol (2014) 592:3937–40. 10.1113/jphysiol.2014.270710 25225252 PMC4198000

[66] Jordan J CrossTalk opposing view: which technique for controlling resistant hypertension? Carotid sinus stimulation. J Physiol (2014) 592:3933–5. 10.1113/jphysiol.2013.268078 25225251 PMC4197999

[67] Kopp UC Cicha MZ Smith LA Dietary sodium loading increases arterial pressure in afferent renal–denervated rats. Hypertension (2003) 42:968–73. 10.1161/01.HYP.0000097549.70134.D8 14568995

[68] Gazdar AF Dammin GJ Neural degeneration and regeneration in human renal transplants. N Engl J Med (1970) 283:222–4. 10.1056/NEJM197007302830502 4912789

[69] Couch NP Mc BR Dammin GJ Murray JE Observations on the nature of the enlargement, the regeneration of the nerves, and the function of the canine renal autograft. Br J Exp Pathol (1961) 42:106–13. 13695911 PMC2082414

[70] Booth LC Nishi EE Yao ST Ramchandra R Lambert GW Schlaich MP Reinnervation of renal afferent and efferent nerves at 5.5 and 11 months after catheter-based radiofrequency renal denervation in sheepnovelty and significance. Hypertension (2015) 65:393–400. 10.1161/HYPERTENSIONAHA.114.04176 25403610

